# Utility of wearable physical activity monitors in cardiovascular disease: a systematic review of 11 464 patients and recommendations for optimal use

**DOI:** 10.1093/ehjdh/ztab035

**Published:** 2021-05-06

**Authors:** Matthew Hammond-Haley, Christopher Allen, Jennie Han, Tiffany Patterson, Michael Marber, Simon Redwood

**Affiliations:** 1 British Heart Foundation Centre of Research Excellence, King's College London, Rayne Institute, St Thomas' Hospital, Westminster Bridge Road, London, SE1 7EP, UK; 2 Department of Cardiology, Guys’ and St Thomas NHS Foundation Trust, St Thomas' Hospital, Westminster Bridge Road, London, UK; 3 Royal Lancaster Infirmary, Ashton Road Lancaster, LA1 4RP, UK

**Keywords:** Physical activity, Wearable activity monitors, Remote outcome monitoring, Cardiovascular disease, accelerometery

## Abstract

**Aims:**

Physical activity (PA) plays an important role in primary and secondary prevention of cardiovascular disease (CVD), functioning as a marker of disease progression and response to therapy. Real-world measurement of habitual PA is now possible through wearable activity monitors, however, their use in cardiovascular patients is not well described.

**Methods and results:**

We performed a systematic review to summarize how wearable activity monitors have been used to measure PA in patients with CVD, with 11 464 patients included across 108 studies. Activity monitors were primarily used in the setting of cardiac rehabilitation (46, 43%). Most often, triaxial accelerometers (70, 65%) were instructed to be worn at the hip (58, 54%) for 7 days (*n* = 54, 50%). Thirty-nine different activity monitors were used, with a range of accelerometer specific settings for collection and reporting of activity data. Activity was reported most commonly as time spent in metabolic equivalent-defined activity levels (49, 45%), while non-wear time was defined in just 16 (15%) studies.

**Conclusion:**

The collecting, processing, and reporting of accelerometer-related outcomes were highly heterogeneous. Most validation studies are limited to healthy young adults, while the paucity of methodological information disclosed renders interpretation of results and cross-study comparison challenging. While accelerometers are promising tools to measure real-world PA, we highlight current challenges facing their use in elderly multimorbid cardiology patients. We suggest recommendations to guide investigators using these devices in cardiovascular research. Future work is required to determine optimal methodology and consensus-based development of meaningful outcomes using raw acceleration data.

## Graphical Abstract

**Figure:**
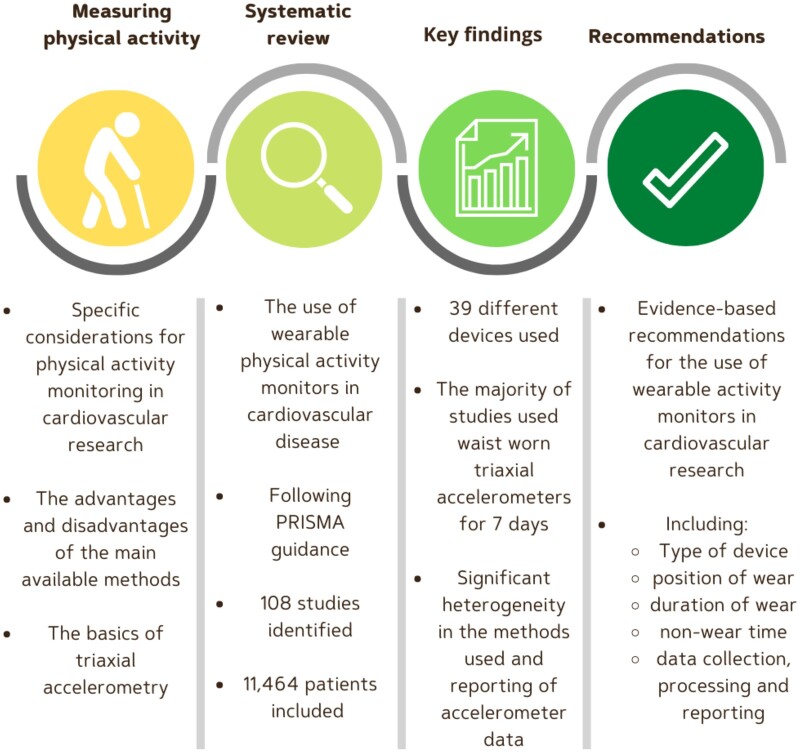

## Introduction

Physical inactivity is the fourth leading cause of death worldwide.^1^^,^^2^ Even modest increases in physical activity (PA) are associated with reduced cardiovascular morbidity and mortality, both in the general population and those with established cardiovascular disease (CVD).^3–6^ Increasing PA is accordingly outlined as an international priority, critical to reducing the global burden of CVD,^7–9^ with many currently falling short of accepted targets.^6^^,^^10^ Moreover, since capacity for PA may be directly limited by the disease process, habitual activity levels may be a useful marker of disease progression or response to specific interventions. Our ability to reliably measure PA in cardiovascular patients is therefore imperative.

Recent years have witnessed a sharp increase the uptake of digital, wearable health technologies, accelerated within the context of the coronavirus disease-2019 (COVID-19) pandemic, where the ability to perform remote clinical assessment safely and accurately has rightly assumed even greater importance.^11^ However, the challenge is to find and select devices with a proven evidence-based efficacy, particularly for the older CVD population; an expanding demographic historically underserved by such technologies.^12^

PA can be defined as ‘any bodily movement produced by skeletal muscles that results in caloric expenditure’.^13^ Total energy expenditure (EE) is influenced by basal metabolic rate (the lowest level of energy expended at rest), which is largely dependent on body mass. It can be estimated that in healthy, normal-weight individuals, resting EE equates to approximately one kilocalorie (kcal)/kg/h.^14^ This basal EE is often described as one metabolic equivalent (MET), with multiples of this unit widely used to express activity intensities. In the general population, moderate intensity PA typically refers to 3.0–5.9 METS and vigorous intensity >6.0 METS.^15^ The primary aim in measuring PA is to quantify the frequency, duration and intensity of activity over a defined period,^14^ permitting estimation of PA-associated EE. A wide range of methods are available for measuring PA, which have evolved rapidly over recent years to reflect technological advancements, particularly miniaturization and connectivity.

**Figure 1:**
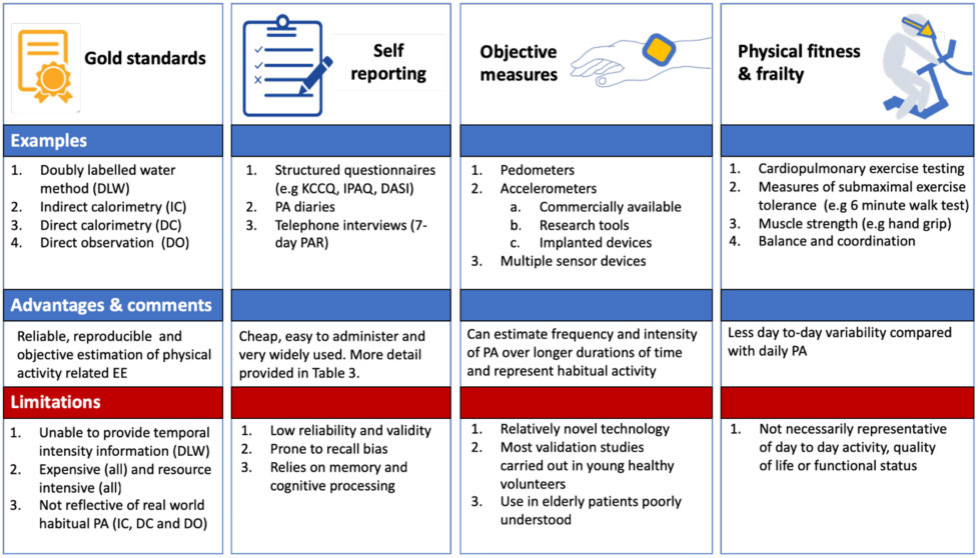
Measuring physical activity and fitness. Gold standard methods to assess physical activity include the ‘doubly labelled water method’ (DLW), indirect and direct calorimetry and direct observation. DLW allows estimation of physical activity related energy expenditure over a period of 10–20 days; however, it is not able to provide temporal intensity information, such as the frequency, intensity and duration of bouts of PA. Indirect calorimetry estimates total energy production by measuring CO_2_ production, while direct calorimetry measures total body heat production. These methods are both generally expensive, laboratory based and therefore not reflective of real-world PA. Direct observation is valuable in gaining detailed information on the types of PA, but has a number of important limitations as shown. Self-reporting of PA is generally cheap and easy to administer, although also has a number of important limitations. PA measurement using wearable devices (including pedometers and accelerometers) has the advantage of permitting objective measurement of PA over longer periods of time in free living subjects. Physical fitness is comprised of cardiorespiratory fitness (CRF), muscle endurance, and flexibility, balance, agility and coordination, with various methods available to test each of these components. Maximal uptake of oxygen during exercise (VO_2max_) can be measured directly, or more commonly estimated from the peak work rate achieved on a cycle or treadmill ergometer. These methods do not necessarily reflect the habitual PA which superimposes lifestyle and motivational factors that act in the free-living state, over and above baseline physical fitness.

**Figure 2:**
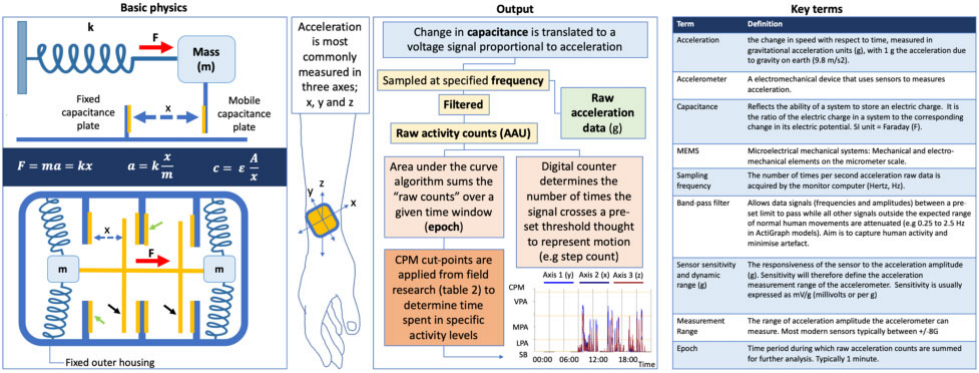
How accelerometers work. Basic physics: Accelerometery can be described in terms of a mass–spring system, operating under Newton’s 2nd law of motion (F = ma) and Hooke’s law (F = kx). When a force (F) (e.g. during PA) displaces the mass (m), the string [which has a known stiffness or ‘spring constant’ (k)] generates a proportional restoring force. When ‘m’ and ‘k’ are known, acceleration (a) can be determined from the resultant displacement (x) using the equations shown. Therefore, acceleration can be indirectly measured by measuring displacement. The bottom panel demonstrates a simplified capacitive accelerometer sensor, with a mobile spring suspended mass with multiple capacitance plates (black arrows) which move as force is applied, and fixed outer housing with stationary capacitance plates (green arrows). As the distance between pairs of plates changes (x), the capacitance changes in an inversely proportional relationship as shown by the third equation, where *c* = capacitance, *ε* = permittivity of the medium between the plates, *A* = area of the conductive plates, and *x* = distance between two plates. Capacitance is converted to a voltage signal proportional to acceleration. Modern day MEMs type sensors use the same principle, with components built and assembled on a micrometre scale. Output: Acceleration data is collected at a specified sampling frequency, typically between 30 and 100 times per second (30–100 Hz). Pre filtered raw acceleration data (g) represents the g-force sampled at a given frequency. This voltage signal can be converted to a digital series of numbers called ‘raw counts’ or arbitrary accelerometer units (AAUs). Exactly how the initial voltage signal is amplified, filtered and processed to produce counts varies between devices. The data is usually filtered using a band-pass filter. Activity counts are then processed in a variety of ways. Most commonly counts are summed over a pre-selected epoch (for raw data, the sampling frequency can be considered the epoch length), which are then used to produce outcomes such as time spent in different activity levels (using cpm cut-offs derived from calibration studies). The bottom graph shows an example of this type of output, showing summed counts per minute over the course of the day, with the yellow lines representing example thresholds for sedentary behaviour (SB), light PA (LPA), moderate PA (MPA) and vigorous PA (VPA).

The various methods to assess PA are summarized in *Figure 1*, and include gold-standard measures of EE,^16–18^ self-reporting (e.g. questionnaires and interviews)^19–22^ and wearable activity monitors (including pedometers and accelerometers). This review focuses on the objective estimation of PA in free living subjects with increasingly small, easily portable wearable devices.^23^ Initially developed as research tools, these devices are increasingly popular in the consumer market.^24^ Capacitive micro-electro mechanical systems (MEMS) type accelerometer sensors have become the cheapest and most widely available in recent years. An overview of the basic physics of accelerometery, capacitive MEMS-type sensors and the generation of accelerometer output data is accordingly provided in *Figure 2*. Accelerometery use has also been extended to implanted devices, including implantable cardiac devices, which lie outside the scope of this review.^25^^,^^26^ Similarly, smartphone technology to assess PA is also rapidly improving and may represent a promising alternative to wearables for both the assessment of PA and as an intervention in improving health outcomes.^27^

The aim of this review was to examine how wearable activity monitors have been used over the last 20 years to measure PA in patients with CVD. This includes the types of devices used, and the methodology employed to collect and report PA data, including the anatomical location of wear, assessment period, collection procedure and data processing and reporting. While the technology will have clearly evolved over this time, this review focused on the wider methodologies adopted, beyond those constrained by device-specific capabilities.

## Methods

A systematic search was performed of PubMed and Embase databases were performed in accordance with PRISMA guidelines (Supplementary material online). We limited our search to studies published from 2001 to present (1 November 2020) to focus on relatively modern technologies. Reference lists of included trials were also screened. Inclusion criteria comprised any original study [including observational studies and randomized control trials (RCTs)] which enrolled patients with heart disease and used wearable activity monitors to measure and report PA data, as either an outcome or intervention. Studies in paediatric populations, adult congenital heart disease, non-cardiology general populations and those monitoring PA by implantable cardiac devices were excluded; as were conference abstracts and review articles.

Two authors (C.A. and M.H.-H.) independently screened and selected suitable studies, and any discrepancies were adjudicated by a third investigator (J.H.). Data were then extracted on the study design, research area, eligibility criteria, activity monitor(s) used, and methods used to gather data on activity, including; position worn, duration of wear, minimum criteria for inclusion of data and excluded time. Data were also extracted on the reporting of activity related outcomes, the thresholds used to categorize activity levels, timing of follow-up activity measurements (if applicable) and the use of any alternative measures of PA (e.g. questionnaires) or cardiorespiratory fitness.

## Results

A total of 1045 studies were identified (after 59 duplicates were removed). After screening for eligibility, 108 studies meeting the inclusion criteria were included, involving 11 464 patients. Details of these studies can be found in the Supplementary material online.

**Figure 3:**
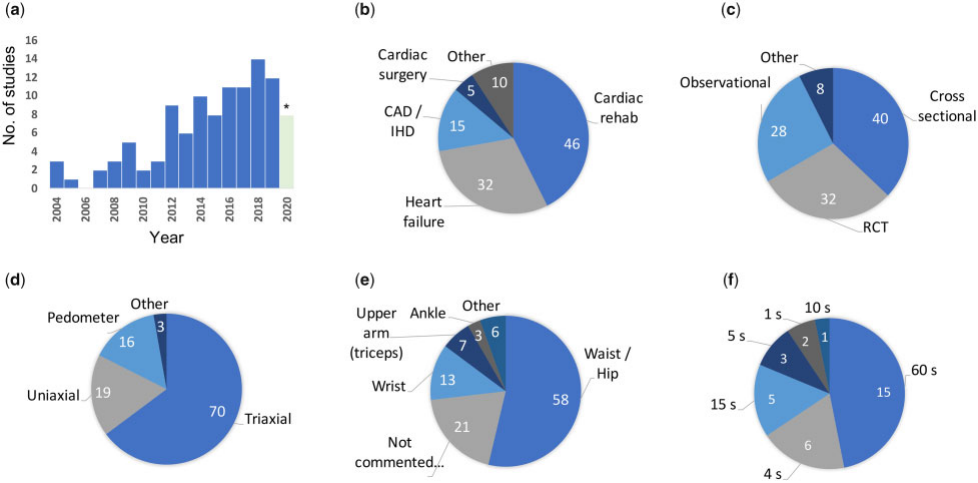
Results. (*A*) Bar chart demonstrating the increase in the number of cardiology studies using wearable activity monitors over the last 16 years. *Up to 1 November 2019. Pie charts demonstrating; (*B*) The number of trials by research area. ‘Other’ included general cardiovascular disease patients (4), inherited cardiac disease/cardiomyopathies (2), LV assist devices (2), Aortic stenosis, pre TAVI (1), cardiac biomarkers (1). (*C*) The design of included trials, (*D*) the types of activity monitors deployed. ‘Other’ included biaxial (2) and omniaxial (1). (*E*) The requested position of wear. ‘Other’ included chest (2), pocket or necklace (1), breast pocket or hip (1), wrist and waist (2). (f) The reported epoch durations in seconds (s).

The oldest study suitable for inclusion was published in 2004. Since then, the number of cardiology studies using activity monitors has increased steadily (*Figure 3A*). The majority of included studies were from the USA (31, 29%), followed by Japan (13, 12%) and Canada (12, 11%). Most were in the setting of cardiac rehabilitation (CR) (46, 43%) or heart failure (32, 30%) (*Figure 3B*). The majority were of cross-sectional design (*n* = 40, 37%), followed by 32 RCTs (30%), and 28 prospective observational studies (26%) (*Figure 3C*). Of the 32 RCTs included, 12 (38%) included an activity monitor as at least part of the intervention (e.g. pedometer-based feedback on daily activity levels in the setting of CR) while in 21 studies (66%) PA was measured as either a primary or secondary outcome.

Thirty-nine different activity monitors were used across the 108 studies, including 36 accelerometers and three pedometers. The ActiGraph GT3x/GT3x+ triaxial accelerometer was the most commonly used, employed in 22 included studies (20%). The majority used triaxial accelerometers (70, 65%) (*Figure 3D*), particularly those published within the last 8 years (2012–2019, 73%), compared to the first 7 years (2004–2011, 32%), in line with technological advancement. Seventeen (44%) of the activity monitors used were consumer products, with the remaining 22 (56%) marketed primarily as research tools. *Table 1* summarizes the accelerometers used in more than one study. Most devices were instructed to be worn at the waist (*n* = 58, 54%), followed by the wrist (*n* = 13, 12%). Notably, a significant proportion of the studies did not comment on where the activity monitor was instructed to be worn (*n* = 21, 19%) (*Figure 3E*).

**Table 1:** Summary of activity monitors used in more than one study

Company, location	Activity monitor model	Year product first released	Commercially available?	Axes	Sensor type	Sample frequency (Hz)	Raw output	Position worn	Number of studies
Actigraph, Florida, USA	GT3x/GT3x+	2009/2010 (GT3x+)	No	Triaxial	Capacitive MEMS	30–100	Yes	Waist/hip	22
	GT1M	2005	No	Uniaxial	Capacitive MEMS	30	Yes	Waist/hip	10
	wGT3x/wGT3X-BT	2012/2013	No	Triaxial	Capacitive MEMS	30–100	Yes	Wrist, waist, ankle, thigh	4
	7164	1995	No	Uniaxial	Piezoelectric	10	No	Waist/hip	4
Suzuken Co, Ltd, Nagoya, Japan	Kenz Lifecorder EX	2007	Yes	Uniaxial	piezoelectric	32	No	Waist/hip	10
BodyMedia, Pennsylvania, USA	Sensewear armband^a^	2001	Yes	Triaxial	MEMS	32	No	Arm (triceps)	7
New-Lifestyles, Inc, Missouri, USA	NL-2000		Yes	Triaxial	piezoelectric			Waist/hip	6
Phillips Respironics, Oregon, USA	Actical			Omni-axial^b^	piezoelectric	32	No	Wrist, Waist/hip, Ankle	2
	Actiwatch II		No	Triaxial	piezoelectric	32		Wrist	2
Omron Healthcare Co. Ltd, Japan	Active Style Pro HJA-750C		Yes	Triaxial				Waist/hip	2
Fitbit Inc, San Francisco, USA	Fitbit Zip	2013	Yes	Triaxial				Waist/pocket/bra	2
Kineteks Corp, Vancouver, British Columbia, Canada	Tractivity		Yes	Uniaxial				Ankle	2

a No longer available. Includes Armband Pro (three studies), Mini (two studies), Armband (one study).

b Although the sensor is omni-directional, it is positioned in such a way that when worn at the hip it is most sensitive to vertical accelerations. Year of product release represents the first year these models were released, with subsequent model upgrades not demonstrated in this table. Older ActiGraph models have been discontinued, with newer ActiGraph models backward compatible with previous models in terms of output.

A pre-defined requested wear period was reported in 99 studies (92%), most commonly 7 days (*n* = 54, 50%); although ranging from 3 h to 3 months. Sixty studies (55%) explicitly outlined periods during which the monitor should not be worn, including; while sleeping (*n* = 35, 32%), bathing or swimming (*n* = 34, 31%) or during medical testing or supervised exercise programmes (*n* = 2, 1%). Four studies (4%) directed participants to wear the monitor continuously without exception, while 48 studies (44%) did not comment on this.

Fifty-five studies (51%) commented on the quality of data required for inclusion in analysis, such as the minimum wear-time per day for each day to be considered valid, and the minimum days worn. The most common criterion for inclusion was wear-time of ≥10 h per day (*n* = 25, 23%), over a minimum of either 4 (*n* = 18, 17%) or 3 days (*n* = 12, 11%). Of these studies, the majority gave no rationale for this approach, although two referenced prior work using similar thresholds,^28^^,^^29^ and only one study referred to recommended PA monitoring quality standards.^30^

Twelve studies (13%) specifically commented on collecting data over a longer period than that used for analysis. For example, five studies (5%) requested devices to be worn for 8 days and only analysed the last 7 days of data (to account for an anticipated reactive increase in activity on first fitting the monitor). Non-wear time definitions were described in just 16 (15%) studies, with significant heterogeneity between methods used; most commonly defined as >60 min of consecutive zero counts, with allowances of one-to-two minutes of counts between 0–100 and 0–150 (*n* = 6, 6%). Alternative definitions of non-wear time included >60 min of consecutive zero counts without exceptions (*n* = 5, 5%), >90 min of consecutive zero counts (*n* = 2, 2%), >90 min of consecutive zero counts with allowances (*n* = 1, 1%), >10 min of consecutive zero counts (*n* = 1, 1%), or <100 steps recorded over 1 day (*n* = 1, 1%).

Six different epoch lengths were used in the included studies, ranging from 1 to 60 s (*Figure 3F*). Activity was most often reported as time spent in MET-defined activity levels (e.g. light, moderate and vigorous PA, most often in min per day) (49 studies, 45%) or average step counts (47 studies, 44%) (*Figure 3G*). Of those reporting time spent in MET-related activity intensity levels, the activity counts per min (cpm) thresholds used were reported in just 29 studies (59%). A marked range of five different cutpoints were used to define the threshold of at least moderate PA (MVPA), varying from 760 cpm to 2690 cpm. These cutpoints and the original studies are described in *Table 2*.

**Table 2:** Summary of the original validation studies for the main activity cutpoints used by the included studies

Author and year	Cutpoints for MVPA (cpm)	Description of study population/method	Number of studies using cut-off
Freedson, 1998^64^	1952	Fifty young healthy volunteers including 25 males with a mean age of 24.8 ± 4.2 years, and 25 females with a mean age of 22.9 ± 3.8 years. Laboratory based graded exercise on a treadmill with measurement of oxygen consumption.	12
Troiano, 2008^65^	2020	Cross-sectional study of 6329 participants with at least 1 day of accelerometer data and 4867 participants with at least four days. Free-living accelerometer data. Heathy, civilian, non-institutionalized U.S. population. Participants categorized into age groups (6–11 years, 12–19 years, 20–59 years, 60+ years). Mean age in the 60+ age group was 71 ± 0.4 years for men and 70.4 ± 0.2 years for women.	5
Sasaki, 2011^53^	2690	Fifty young healthy adult volunteers (28 males, 22 females), with a mean age of 26.9 ± 7.7 years, wore the GT3X and GT1M accelerometers and completed a graded exercise treadmill protocol.	6
Matthews, 2005^38^	760	Cross-validation of laboratory-based study^66^ including 81 participants aged 19–74 years, and a field-based ‘free living’ study,^67^ with ten participants including 4 men with a mean age of 26.7 years, and 6 women with a mean age of 26.5 years.	4
Copeland, 2009^a68^	1040	Healthy older population (*N* = 38, mean age 69.7 ± 3.5 years), completed a laboratory-based calibration	2

a The only study to focus on an older population, while the other validation studies were all carried out in healthy young volunteers.

Other reported outcome measures included estimated mean EE (kcals) (*n* = 22, 20%), total raw activity counts (*n* = 18, 16%) and time spent in sedentary behaviour (min/d) and/or number of sedentary bouts (*n* = 14, 13%). Wear-time was included as a secondary outcome measure in 12 studies (11%). Fifty-seven studies (53%) measured activity during a one-off period, whilst the remaining 51 (47%) studies measured activity at baseline and during one or more follow-up periods, between 2 weeks and 12 months.

Self-reported activity measures were used alongside wearable activity monitors in 49 of the included studies (45%). These included activity logs (*n* = 13, 12%), and activity-related questionnaires, such as the International Physical Activity Questionnaire (IPAQ) (*n* = 6, 6%), the 7-day Physical Activity Recall (7-day PAR) (*n* = 5, 5%) and the Kansas City Cardiomyopathy Questionnaire (KCCQ) (*n* = 3, 3%). Questionnaires used in more than one study are summarized in *Table 3*. Sixty-six studies (60%) also measured markers of cardiorespiratory fitness, which included graded cardiopulmonary exercise testing to estimate VO_2 max_ (*n* = 28, 26%), the 6MWT (*n* = 20, 19%) and hand-grip strength (*n* = 5, 5%). Measures of physical fitness used more than once are summarized in *Table 4*.

**Table 3:** Summary of the main subjective measures of physical activity in the included studies.

Questionnaire	Description	Number of studies using questionnaire
IPAQ^69^	Short and long forms available. Designed specifically for adults (18 – 65 years old). Four main domains: (i) transportation, (ii) work, (iii) household and gardening tasks, and (iv) leisure time, including exercise and sport participation. Estimate of duration and frequency of PA, with scores given for each category. Designed to be easily adapted in many languages and countries. Versions have been developed for specific populations (e.g. youth, elderly, and foreign language speakers).	6
7-day PAR^70^^,^^71^	Duration of various PA levels/activities (Sleep, Moderate PA, Hard PA. Very hard PA) over the past week are used to estimate EE. Calculations assume that unaccounted-for time was spent in light activity.	5
Kansas city cardiomyopathy questionnaire^72^	Aims to quantify physical limitations, symptoms, self-efficacy, social interference and quality of life.	3
Godin leisure time exercise questionnaire^73^	Initially developed for youth. Estimates mild, moderate and strenuous activity in leisure time over a ‘typical week’.	3
Borg's rating of perceived exertion^74^^,^^75^	Widely used psycho-physical tool to assess subjective perception of effort during exercise. Data revealing the relationships and validity of RPE compared to objectively assessed metabolic criterion is lacking.	3
Duke activity severity index^76^	12-item scale on the ability to do increasingly strenuous activities, from which a fitness score estimating VO_2max_ can be calculated	2
Active Australia Survey^77^	Developed for adults. Measured over last 7 days or usual week. Walking briskly, moderate leisure activity, vigorous leisure activity	2

**Table 4:** Summary of the main surrogate measures of physical fitness used in the included studies

CP fitness measure	Description	Number of studies
CPET^78^	Provides dynamic overall assessment of the response to exertion involving the pulmonary, cardiovascular, haematopoietic, neuropsychological, and skeletal muscle systems. It enables measurement of exercise performance and functional capacity, largely through the measurement of VO_2 max_.^78^	28
6MWT^79^	An alternative to CPET as a measure of functional exercise capacity. A simple test which requires a 100-ft hallway but no specific equipment or advanced training. It measures the distance that an individual can walk on a flat, hard surface in six minutes. As with CPET it provides an overview of the integrated responses of all the systems involved during exercise, however no detailed information about the different systems involved (e.g. VO_2max_) and assesses sub-maximal exercise capacity.	20
Handgrip strength^80^	Grip strength is a simple measurement can be assessed quantitatively using a hand dynamometer. It is a widely used measurement of muscle strength (a component of physical fitness), particular in older patients in the context of sarcopenia, frailty and undernutrition.	5
Gait speed^81^	An alternative quick, inexpensive, reliable measure of functional capacity. Mainly utilized in older adult populations, it is widely used as a screening tool for a wide range of health outcomes including hospitalizations, nursing home placements, mortality, poor quality of life and physical and cognitive decline.^81^ As with the 6MWT, no specialized equipment or training is required.	3

CPET, cardiopulmonary exercise testing; 6MWT, 6-min walk test. Other methods used included knee flexion and extension testing (*n* = 2), balance testing (*n* = 2), chair stand test (*n* = 2), and the Short Physical Performance Battery (*n* = 2)—a composite measure which includes the results of gait speed, chair stand and balance tests.

## Discussion

In this systematic review, we broadly evaluated the use of wearable PA monitors in cardiovascular research. This included the types of devices being used, the decisions and reporting of key methodological details such as the position and duration of wear, device-specific settings, processing and reporting of PA data. This was the first comprehensive systematic review of this nature on wearable activity monitors in patients with CVD, and as such consensus is lacking on how researchers should utilize this technology in this patient population.

Most included studies were in the context of heart failure or CR. Cardiac rehabilitation refers to specific exercise-based programmes aiming to improve PA in patients with established CVD, which improve outcomes in coronary heart disease and heart failure.^31^^,^^32^ Objectively measuring PA (both during CR-specific activities and everyday life) in these populations is therefore important, with most studies showing target PA levels are not being met. In addition, a number of CR studies have used wearable activity monitors as part of the intervention itself, aiming to increase PA either by allowing self-monitoring of daily step counts as a motivator to increase activity or providing individualized feedback based on activity monitor data.^33^^,^^34^

Outside the CR and heart failure settings, a relatively small number of studies were identified in coronary artery disease, with minimal use elsewhere. There is clear scope to extend the use of activity monitors given the role of habitual PA as a prognostic marker in a wide range of other cardiovascular conditions.^4^ This is of particular importance currently, due to the ongoing effects of COVID-19 pandemic on healthcare research, with efforts needed to adapt RCT methodology including remote endpoint assessment.^11^

The ActiGraph GT3x was the most commonly used activity monitor, although there was considerable temporal and geographical variation. There has been a shift towards triaxial accelerometery as this technology, which is thought to improve estimates of EE compared to uniaxial devices, has become more affordable and widely available.^35^ There is no convincing data to suggest a preference between commercially available or research grade devices,^36^ particularly in older patients. Depend on the specific question being asked, a trade-off needs to be considered between raw data from research grade devices and the scale of data achievable by low-cost consumer devices.

Position of accelerometer wear significantly affects PA measurement, with some estimates suggesting wrist compared with waist-worn devices overestimate PA by as much as 20% to 40%.^37^ Waist worn accelerometers have traditionally been considered to provide the most accurate measurement of centre of mass acceleration,^37^ and in keeping with this our review found accelerometers were most often instructed to be worn at the waist. However, it is worth noting that accelerometers do not generally provide qualitative information on the type of PA being undertaken, and activities such as cycling and rowing may be underestimated by waist worn accelerometers.^37^

Similarly, common daily activities which expend energy due to muscle contractions, without much physical motion (e.g. housework), will be underestimated by a single activity monitor worn at the waist.^37^ While in healthy individuals EE from dynamic activities accounts for the major source of overall PA,^38^ in elderly patients with multiple comorbidities this approach may lead to underestimation of total habitual PA. In addition, with the emergence of commercially available wrist worn tri-axial accelerometers, wear time and compliance may be greater compared with waist-worn devices.^37^ Most studies comparing wear position have been in young healthy volunteers with no clear consensus on the optimal site for elderly participants with low habitual PA. Furthermore, while using multiple accelerometers may slightly improve estimates of PA, this may not warrant the associated increased subject burden.^39^

Accelerometers were most often instructed to be worn for 7 days during waking hours and removed during water-based activities. A valid week was most often defined as a minimum of four valid days, with a valid day defined by at least 10 h of wear-time, consistent with previous findings.^40^ Seven days is the traditionally adopted period for habitual PA accelerometery measurement, chosen to account for expected inter-day variability in free-living subjects, thought to be influenced by both internal and external factors including the weather and day of the week.^41^

In a study of 475 middle-aged Irish adults, participants were most sedentary on weekends, with the authors suggesting that 6 days of monitoring, including Sunday, was needed to reliably capture weekly habitual activity.^42^ By contrast, although a similar inter-day variation was observed elsewhere, the authors concluded the difference between days was unlikely to be of practical importance and that any 3 days could provide a sufficient estimate.^43^ A number of included studies specified a weekend day must be included in the collection period, apparently to adjust for this ‘weekend effect’. However, in a cohort of colorectal cancer patients no significant differences were observed between weekdays and weekend days,^44^ with similar findings in children.^45^ Furthermore, based on analysis of a large population of US adult accelerometery data, Wolff-Hughes *et al*.^46^ suggested that non-random inclusion of weekend days introduced bias to group-level activity estimates and should be avoided.

These conflicting findings likely represent the heterogeneity between the populations being studied, suggesting that standard protocols should be tailored for specific patient groups. For example, in older retired populations with activity-limiting conditions, it is possible that less inter-day variability in habitual PA will be observed, with a less pronounced ‘weekend effect’, hence shorter collection periods not including the weekend may be sufficient. This decision will be influenced by the level of precision required based on the research question, ranging from drawing conclusions from estimates of mean activity in a large group, to ranking individuals within a group or tracking individual changes in PA for instance pre- and post- intervention.^41^ If required, precision can be improved by increasing the duration of wear or by increasing the number of participants.

Long continuous accelerometer zero counts could represent either non-wear or inactivity, which can be difficult to distinguish. The issue of defining non-wear was discussed in surprisingly few (15%) of the included studies, with significant heterogeneity in methods described. Correct differentiation is particularly important in studies focusing on sedentary behaviour or inactivity, and log-book or activity diaries can be helpful in successfully identifying these periods. In the included studies, there was little justification for the choice of protocol, and it is possible that, as with choosing a duration of wear, non-wear time cutpoints should be tailored to specific groups. For example, older multimorbid patients may be more likely to sit inactive for longer periods than younger adults, and this could be incorporated into methodology to better define non-wear time in this group (e.g. a longer period of consecutive zero counts). This issue requires further investigation, and protocols used should be clearly reported in future studies.

Significant heterogeneity was observed in the technical aspects of accelerometer methodology employed, including the sampling frequency and bandwidth filter settings of the included devices (*Table 1*). Most modern widely used accelerometers have a default sampling frequency of 30 Hz, but many allow programming during initialization between 30 and 100 hz. Choosing an appropriate rate of data acquisition is thought to be important to ensure the full range of human motion is captured,^47^ and affects PA measurements,^48^ However, the impact of such settings on PA outcomes, particularly in the elderly, is poorly understood and warrants further investigation. Bandpass-filter settings are often proprietary information and seldom fully disclosed, although also impact final activity-related measurements.^47^ Similarly, a wide range of epoch lengths were used in the included studies; a feature known to significantly affect PA measurement outputs.^49^ One-minute epochs have traditionally been the standard length and this was the most commonly reported in our review. Consensus is lacking as to how accelerometers should best be initialized to reliably collect habitual PA data in older adults.^50–52^

Significant heterogeneity was also observed in the methods of processing accelerometer data and reporting outcomes. Most notable was the range of thresholds used to categorize activity counts into MET-related activity intensity levels, with the minimum threshold for MVPA ranging from >760 cpm^38^ to >2690 cpm.^53^ As such, until consensus is reached, caution must be exercised when comparing studies reporting outcomes such as minutes spent in a particular activity intensity level. Furthermore, these cutpoints were largely derived from studies in healthy adult volunteers (*Table 2*) and may not be appropriate for all populations. Specific thresholds in the elderly have been suggested taking into account VO_2max_, gender and accelerometer-axis for more accurate assessment of relative PA intensity.^54^ Similarly, intensity cutpoints based on an individual’s fitness capacity may more accurately reflect PA level compared to thresholds based on arbitrary absolute activity count cutpoints.^51^^,^^55^^,^^56^ Until consensus is reached in older multimorbid populations, it may therefore be more helpful to focus on continuous variables such as step counts and raw acceleration data as discussed below.

As discussed, most validation work in objective PA measurement has been performed in young healthy volunteers, therefore the move towards methodological consensus in cardiovascular and elderly multimorbid patient populations in general is limited by a number of key knowledge gaps. As the use of accelerometers in these patient groups is expanding, further work is indicated to explore such issues, including, but not limited to, the impact of accelerometer position, duration of wear, non-wear time definitions and technical accelerometer specifications and setup on habitual PA and EE estimates. It follows that until consensus is reached, collection and reporting of raw activity counts is desirable to ameliorate some of these issues and allow a degree of cross-study comparison.^57^ It must however be noted that even raw accelerometer counts may be influenced by accelerometer-specific and investigator-controlled settings such as sampling frequency and epoch length, and hence still require reporting and critical analysis when being compared across studies. One of the main challenges currently is the difficulty in gaining meaningful insight into habitual PA from raw acceleration data. A consensus approach to analysis methods adopting algorithms capable of processing raw data signals into clinically relevant outcomes is therefore highly desirable.^57^ While examples of tri-axial raw data analytical approaches already exist,^58^ our review demonstrates they are yet to be widely adopted by the cardiovascular research community.

Combining accelerometery and other wearable biosensor data, such as heart rate monitoring, body temperature, blood pressure and galvanic skin response is possible with a number of commercially available and research-grade devices. However, since only two of the 39 activity monitors used in the included studies had this additional capability, assessment of these technologies was outside the scope of this review. Incorporating heart-rate data may yield more precise estimates of EE than accelerometery alone,^59^ although limitations to this approach include patients in whom heart rate is pharmacologically limited or device controlled (e.g. in the presence of beta-blockade or a pacemaker), unless specific EE estimation models are applied.^60^ Additionally, to our knowledge the presence of common arrhythmias, such as atrial fibrillation, on the estimation of EE has not yet been investigated.

There is clear scope for wearable activity monitors to play a larger role in objective PA monitoring in the clinical setting. For example, the New York Heart Association classification is used for functional assessment of patients with heart failure. Although widely used, this relies on patient recollection of activity and individual application by clinicians, and correlates poorly with formally measured exercise capacity,^61^ and more objective measures may be useful for disease classification to guide management and prognostication. Furthermore, in the setting of severe aortic stenosis, development of symptoms (increasing shortness of breath on exertion, resulting in a reduction in exercise capacity, and syncope) marks a significant increase in risk of mortality and the need for intervention by surgical or transcatheter valve replacement. Objectively measured PA may play a role in identifying these patients at an earlier stage, complementing patient reporting of activity.

This review was the first to give a systematic and detailed overview of the use of wearable activity in cardiovascular research in general. A number of key limitations should be noted. Firstly, both commercially available and research grade wearable activity monitors continue to evolve with new products released each year, and therefore findings from studies earlier in the collection period may be less relevant to researchers today. However, while the technology has changed over the collection period, the precise issue of heterogeneity in methodology has been constant throughout and remains critical when comparing studies conducted in recent years. Similarly, smartphones have emerged as another widely available tool to monitor PA, although are not technically ‘wearable’ activity monitors and were therefore outside the scope of this review. Although allowing participants to use their own devices may represent an enticing option for researchers, similar issues with standardization of methodology, validation and cross-device compatibility will likely arise.

## Recommendations for optimal use of physical activity monitors in cardiovascular disease

**Figure 4:**
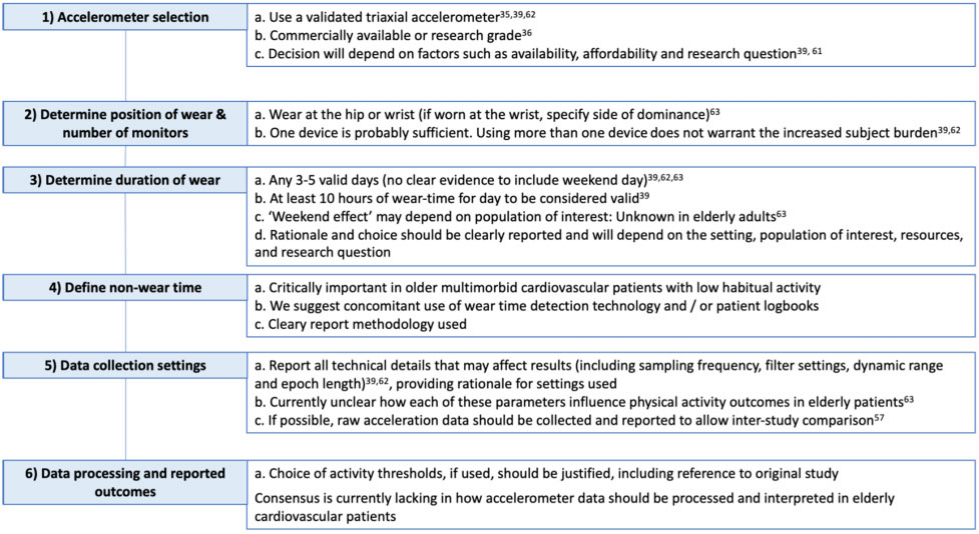
Recommendations for PA measurement in elderly patients. Recommendations for the collection and reporting of physical activity data in older adults, based on previous guidance,^39^^,^^57^^,^^58^ adapted to reflect the limited knowledge base in elderly multi-morbid patients with low habitual PA.

We propose the following recommendations (*Figure 4*) to aid physicians and researchers in designing studies in which habitual PA will be objectively measured, highlighting the minimum methodological detail required to allow meaningful interpretation of results and appropriate inter-study comparison. These recommendations are in-keeping with previous guidance,^39^^,^^62^^,^^63^ yet focus specifically on elderly, multimorbid patients and reflect recent advances in accelerometer technology.

## Conclusions

Both commercially available and research-grade wearable activity monitors have been widely used over the last 30 years in cardiovascular research, particularly in the setting of heart failure and cardiac rehab, with much wider potential application. A wide range of devices have been used, predominantly using triaxial accelerometer technology. Actigraph research-grade devices were most commonly used, and most commonly instructed to be worn at the hip for 7 days. However, we identified significant heterogeneity in the methods used to collect and report PA data in patients with CVD and as a result, direct comparison of PA data between studies is currently extremely challenging, often impossible. Validation studies have predominantly been restricted to young healthy volunteers, with methodological consensus lacking in elderly multimorbid patients. We suggest simple recommendations based on currently available evidence for the reliable and practical measurement and reporting of PA in cardiovascular patients. Future work is critical to answer key remaining questions in this patient population, including optimal position and duration of wear, definition of non-wear time, and consensus-based development of meaningful outcomes using raw acceleration data.

## Supplementary material

Supplementary material is available at *European Heart Journal is available at* online.

## Supplementary Material

ztab035_Supplementary_Material
