# Supplementary material for: Utility of wearable physical activity monitors in cardiovascular disease: a systematic review of 11 464 patients and recommendations for optimal use
Source: Eur Heart J Digit Health. 2021 May 6;2(2):231–43. doi: 10.1093/ehjdh/ztab035 (PMC9707885; doi:10.1093/ehjdh/ztab035)
Supplement: ztab035_Supplementary_Material [file ztab035_supplementary_material.docx]

**Supplementary material**

**
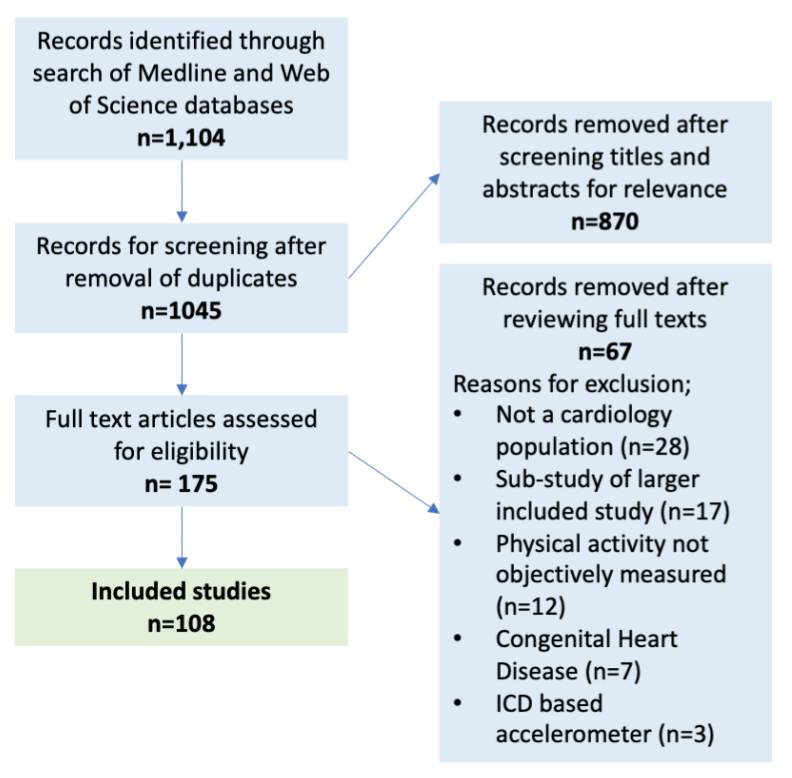
**

**Supplementary Figure 1. PRISMA 2009 Flow Diagram**

Preferred Reporting Items for Systematic Reviews and Meta-Analyses (PRISMA) Flow Diagram showing the process of identifying, screening, and selecting the studies included in this review. Of 1045 studies initially identified, 108 studies met the inclusion criteria.

**Literature search strategy**

Database: PubMed and Embase databases, 2001 to present (November 1st, 2020). Titles and abstracts were searched for the following terms:

‘Physical activity’ AND (‘activity monitor’ OR ‘activity monitors’ OR ‘actigraph’ OR ‘actiwatch’ OR ‘RT3’ OR ‘actiheart’ OR ‘single axis’ OR ‘accelerometer’ OR ‘accelerometry’ OR ‘pedometer’) AND (‘heart’ OR ‘cardiology’ OR ‘cardiac’ OR ‘myocardial’)

**Supplementary Table 1. Details of the 108 included studies**

| First author | Year |
| --- | --- |
| Trecarten^1^ | 2020 |
| Dibben^2^ | 2020 |
| Vetrovsky^3^ | 2020 |
| Ozemek^4^ | 2020 |
| Freene^5^ | 2020 |
| Schmidt^6^ | 2020 |
| Claes^7^ | 2020 |
| Freene^8^ | 2020 |
| Abedalmajeed Shajrawi^9^ | 2019 |
| Okwose^10^ | 2019 |
| Strauch^11^ | 2019 |
| Moreno-Suarez^12^ | 2019 |
| Willis^13^ | 2019 |
| Huffman^14^ | 2019 |
| Andreae^15^ | 2019 |
| Lelis^16^ | 2019 |
| Ramadi^17^ | 2019 |
| Kurose^18^ | 2019 |
| Moghei^19^ | 2019 |
| Deka^20^ | 2019 |
| Hoffmann^21^ | 2018 |
| OrDonnell^22^ | 2018 |
| Sweeting^23^ | 2018 |
| Biswas^24^ | 2018 |
| Parsons^25^ | 2018 |
| Pozehl^26^ | 2018 |
| Strauch^27^ | 2018 |
| Freene^28^ | 2018 |
| Miyahara^29^ | 2018 |
| Ter Hoeve^30^ | 2018 |
| Taherzadeh^31^ | 2018 |
| Saunders^32^ | 2018 |
| Floegel^33^ | 2018 |
| Floegel^34^ | 2018 |
| Shen^35^ | 2017 |
| Mungovan^36^ | 2017 |
| Sweeting^37^ | 2017 |
| Salmoirago-Blotcher^38^ | 2017 |
| McCarthy^39^ | 2017 |
| Ter Hoeve^40^ | 2017 |
| Christle^41^ | 2017 |
| Thorup^42^ | 2017 |
| Waring^43^ | 2017 |
| Vogel^44^ | 2017 |
| Nakajima^45^ | 2017 |
| Alharbi^46^ | 2016 |
| Ramadi^47^ | 2016 |
| Midence^48^ | 2016 |
| Edwards^49^ | 2016 |
| Loprinzi^50^ | 2016 |
| Melin^51^ | 2016 |
| Charman^52^ | 2016 |
| Prince^53^ | 2016 |
| Granegger^54^ | 2016 |
| Thorup^55^ | 2016 |
| Kraal^56^ | 2016 |
| Izawa^57^ | 2015 |
| Alosco^58^ | 2015 |
| Alosco^59^ | 2015 |
| Redfield^60^ | 2015 |
| Frederix^61^ | 2015 |
| Frederix^62^ | 2015 |
| Tang^63^ | 2015 |
| Nishitani-Yokoyama^64^ | 2015 |
| Dontje^65^ | 2014 |
| Devi^66^ | 2014 |
| Evenson^67^ | 2014 |
| Fulcher^68^ | 2014 |
| Oliveira^69^ | 2014 |
| Alosco^70^ | 2014 |
| Byun^71^ | 2014 |
| Borland^72^ | 2014 |
| Izawa^73^ | 2014 |
| Klompstra^74^ | 2014 |
| da Silva^75^ | 2013 |
| Hu^76^ | 2013 |
| Bäck^77^ | 2013 |
| Kaminsky^78^ | 2013 |
| Mohri^79^ | 2013 |
| Izawa^80^ | 2013 |
| Sherwood^81^ | 2012 |
| Karjalainen^82^ | 2012 |
| Alosco^83^ | 2012 |
| Izawa^84^ | 2012 |
| Houle^85^ | 2012 |
| Guiraud^86^ | 2012 |
| Izawa^87^ | 2012 |
| Reid^88^ | 2012 |
| Reid^89^ | 2012 |
| Pinto^90^ | 2011 |
| Ribeiro^91^ | 2011 |
| Houl^92^ | 2011 |
| Tai^93^ | 2010 |
| Toth^94^ | 2010 |
| Barmason^95^ | 2009 |
| Stevenson^96^ | 2009 |
| Brändström^97^ | 2009 |
| Butler^98^ | 2009 |
| Maurer^99^ | 2009 |
| Oliveira^100^ | 2008 |
| Papaspyros^101^ | 2008 |
| Ayabe^102^ | 2008 |
| Hughes^103^ | 2007 |
| Jones^104^ | 2007 |
| Evangelista^105^ | 2005 |
| van den Berg-Emons^106^ | 2004 |
| Izawa^107^ | 2004 |
| Ayabe^108^ | 2004 |

**Reference List of included studies**

1. Trecarten N, Kirkland S, Rainham D, Giacomantonio N, McGowan E, Murnaghan D, et al. Location-Based Sedentary Time and Physical Activity in People Living With Coronary Artery Disease. J Cardiopulm Rehabil Prev [Internet]. 2020 Sep 16 [cited 2020 Nov 23];Publish Ahead of Print. Available from: <https://journals.lww.com/10.1097/HCR.0000000000000533>

2. Dibben GO, Gandhi MM, Taylor RS, Dalal HM, Metcalf B, Doherty P, et al. Physical activity assessment by accelerometry in people with heart failure. BMC Sports Sci Med Rehabil [Internet]. 2020 Aug 12 [cited 2020 Nov 23];12. Available from: https://www.ncbi.nlm.nih.gov/pmc/articles/PMC7425563/

3. Vetrovsky T, Frybova T, Gant I, Semerad M, Cimler R, Bunc V, et al. The detrimental effect of COVID-19 nationwide quarantine on accelerometer-assessed physical activity of heart failure patients. ESC Heart Fail. 2020;7(5):2093–7.

4. Ozemek C, Strath SJ, Riggin K, Harber MP, Imboden MT, Kaminsky LA. Pedometer Feedback Interventions Increase Daily Physical Activity in Phase III Cardiac Rehabilitation Participants. J Cardiopulm Rehabil Prev. 2020 May;40(3):183–188.

5. Freene N, Borg S, McManus M, Mair T, Tan R, Davey R, et al. Comparison of device-based physical activity and sedentary behaviour following percutaneous coronary intervention in a cohort from Sweden and Australia: a harmonised, exploratory study. BMC Sports Sci Med Rehabil. 2020;12:1–8.

6. Schmidt C, Santos M, Bohn L, Delgado BM, Moreira-Gonçalves D, Leite-Moreira A, et al. Comparison of questionnaire and accelerometer-based assessments of physical activity in patients with heart failure with preserved ejection fraction: clinical and prognostic implications. Scand Cardiovasc J. 2020 Mar 3;54(2):77–83.

7. JMIR - Feasibility, Acceptability, and Clinical Effectiveness of a Technology-Enabled Cardiac Rehabilitation Platform (Physical Activity Toward Health-I): Randomized Controlled Trial | Claes | Journal of Medical Internet Research [Internet]. [cited 2020 Nov 23]. Available from: https://www.jmir.org/2020/2/e14221/

8. Freene N, McManus M, Mair T, Tan R, Davey R. High sedentary behaviour and low physical activity levels at 12 months after cardiac rehabilitation: A prospective cohort study. Ann Phys Rehabil Med. 2020 Jan 1;63(1):53–8.

9. Impacts of Treatment Modalities on Physical Activity After F... : Dimensions of Critical Care Nursing [Internet]. [cited 2020 Nov 23]. Available from: https://journals.lww.com/dccnjournal/Fulltext/2019/11000/Impacts_of_Treatment_Modalities_on_Physical.2.aspx?casa_token=t5QHnN1bg8AAAAAA:Ln_3Sn49LxvZh2QvkE3YsHVi4quSChEOpXIF9N0uB44_iip9EI3it6dAcNMdMUDzGmdjZ93YHBoZwx_Uq0gkL2sw2Jg

10. Okwose NC, Avery L, O’Brien N, Cassidy S, Charman SJ, Bailey K, et al. Acceptability, Feasibility and Preliminary Evaluation of a Novel, Personalised, Home-Based Physical Activity Intervention for Chronic Heart Failure (Active-at-Home-HF): a Pilot Study. Sports Med - Open. 2019 Nov 27;5(1):45.

11. Strauch S, Hagströmer M, Bäck M. Objectively Assessed Physical Activity in the Oldest Old Persons With Coronary Artery Disease. J Geriatr Phys Ther 2001. 2018 Jul 3;

12. Moreno-Suarez I, Liew S, Dembo LG, Larbalestier R, Maiorana A. Physical Activity Is Higher in Patients with Left Ventricular Assist Device Compared with Chronic Heart Failure. Med Sci Sports Exerc. 2020 Jan;52(1):1–7.

13. Willis LH, Slentz CA, Johnson JL, Kelly LS, Craig KP, Hoselton AL, et al. Effects of Exercise Training With and Without Ranolazine on Peak Oxygen Consumption, Daily Physical Activity, and Quality of Life in Patients With Chronic Stable Angina Pectoris. Am J Cardiol. 2019 Sep 1;124(5):655–60.

14. Huffman JC, Feig EH, Millstein RA, Freedman M, Healy BC, Chung W-J, et al. Usefulness of a Positive Psychology-Motivational Interviewing Intervention to Promote Positive Affect and Physical Activity After an Acute Coronary Syndrome. Am J Cardiol. 2019 Jun 15;123(12):1906–14.

15. The relationship between physical activity and appetite in patients with heart failure: A prospective observational study - Christina Andreae, Kristofer Årestedt, Lorraine Evangelista, Anna Strömberg, 2019 [Internet]. [cited 2020 Nov 23]. Available from: https://journals.sagepub.com/doi/full/10.1177/1474515119836567?casa_token=8MlMJsDNxyQAAAAA%3AwPIF7Dysgjp3B1mHfzbbLsmP14D9nxrATMx_GHNyLHCex1C6qQyHM5FKdHKUAnxVmLrpAmI2Mg

16. Validity of the Incremental Shuttle Walk Test to Assess Exer... : Journal of Cardiopulmonary Rehabilitation and Prevention [Internet]. [cited 2020 Nov 23]. Available from: <https://journals.lww.com/jcrjournal/Fulltext/2019/05000/Validity_of_the_Incremental_Shuttle_Walk_Test_to.13.aspx?casa_token=Bv1Awu4os3UAAAAA:eU2w803we46r3wDOR9hlMY1vqPzRAEPoyjU7UgW-FIWc4KWLk8JLQnEdcN7WNHVlbuOvEKvAtx9wPdRfthR_kdsDZWU>

17. Ramadi A, Haennel RG. Sedentary behavior and physical activity in cardiac rehabilitation participants. *Heart Lung J Acute Crit Care*. 2019;48(1):8-12. doi:10.1016/j.hrtlng.2018.09.008

18. Kurose S, Miyauchi T, Yamashita R, et al. Association of locomotive activity with sleep latency and cognitive function of elderly patients with cardiovascular disease in the maintenance phase of cardiac rehabilitation. *J Cardiol*. 2019;73(6):530-535. doi:10.1016/j.jjcc.2018.12.015

19. Moghei M, Reid RD, Wooding E, et al. A Longitudinal Examination of the Social-Ecological Correlates of Exercise in Men and Women Following Cardiac Rehabilitation. *J Clin Med*. 2019;8(2). doi:10.3390/jcm8020250

20. Deka P, Pozehl B, Williams MA, Norman JF, Khazanchi D, Pathak D. MOVE-HF: An internet-based pilot study to improve adherence to exercise in patients with heart failure. *Eur J Cardiovasc Nurs J Work Group Cardiovasc Nurs Eur Soc Cardiol*. 2019;18(2):122-131. doi:10.1177/1474515118796613

21. Hoffmann JM, Hellwig S, Brandenburg VM, Spaderna H. Measuring Fear of Physical Activity in Patients with Heart Failure. *Int J Behav Med*. 2018;25(3):294-303. doi:10.1007/s12529-017-9704-x

22. OrDonnell J, Velardo C, Shah SA, et al. Physical Activity and Sleep Analysis of Heart Failure Patients using Multi-sensor Patches. *Conf Proc Annu Int Conf IEEE Eng Med Biol Soc IEEE Eng Med Biol Soc Annu Conf*. 2018;2018:6092-6095. doi:10.1109/EMBC.2018.8513594

23. Sweeting J, Ingles J, Ball K, Semsarian C. Daily Step Count as a Simple Marker of Disease Severity in Hypertrophic Cardiomyopathy. *Heart Lung Circ*. 2018;27(6):752-755. doi:10.1016/j.hlc.2017.12.012

24. Biswas A, Oh PI, Faulkner GE, Alter DA. A prospective study examining the influence of cardiac rehabilitation on the sedentary time of highly sedentary, physically inactive patients. *Ann Phys Rehabil Med*. 2018;61(4):207-214. doi:10.1016/j.rehab.2017.06.003

25. Parsons TJ, Sartini C, Welsh P, et al. Objectively measured physical activity and cardiac biomarkers: A cross sectional population based study in older men. *Int J Cardiol*. 2018;254:322-327. doi:10.1016/j.ijcard.2017.11.003

26. Pozehl BJ, Mcguire R, Duncan K, et al. Accelerometer-Measured Daily Activity Levels and Related Factors in Patients With Heart Failure. *J Cardiovasc Nurs*. 2018;33(4):329-335. doi:10.1097/JCN.0000000000000464

27. Strauch S, Hagströmer M, Bäck M. Objectively Assessed Physical Activity in the Oldest Old Persons With Coronary Artery Disease. *J Geriatr Phys Ther 2001*. July 2018. doi:10.1519/JPT.0000000000000204

28. Freene N, McManus M, Mair T, Tan R, Davey R. Objectively Measured Changes in Physical Activity and Sedentary Behavior in Cardiac Rehabilitation: A PROSPECTIVE COHORT STUDY. *J Cardiopulm Rehabil Prev*. 2018;38(6):E5-E8. doi:10.1097/HCR.0000000000000334

29. Miyahara S, Fujimoto N, Dohi K, et al. Postdischarge Light-Intensity Physical Activity Predicts Rehospitalization of Older Japanese Patients With Heart Failure. *J Cardiopulm Rehabil Prev*. 2018;38(3):182-186. doi:10.1097/HCR.0000000000000296

30. Ter Hoeve N, Sunamura M, Stam HJ, et al. Effects of two behavioral cardiac rehabilitation interventions on physical activity: A randomized controlled trial. *Int J Cardiol*. 2018;255:221-228. doi:10.1016/j.ijcard.2017.12.015

31. Taherzadeh G, Reid RD, Prince SA, et al. Amount and Socio-Ecological Correlates of Exercise in Men and Women at Cardiac Rehabilitation Completion. *Am J Phys Med Rehabil*. 2018;97(11):816-824. doi:10.1097/PHM.0000000000000972

32. Saunders C, Huta V, Sweet SN. Physical Activity, Well-Being, and the Basic Psychological Needs: Adopting the SDT Model of Eudaimonia in a Post-Cardiac Rehabilitation Sample. *Appl Psychol Health Well-Being*. 2018;10(3):347-367. doi:10.1111/aphw.12136

33. Floegel TA, Dickinson JM, DerAnanian C, McCarthy M, Hooker SP, Buman MP. Association of Posture and Ambulation With Function 30 Days After Hospital Discharge in Older Adults with Heart Failure. *J Card Fail*. 2018;24(2):126-130. doi:10.1016/j.cardfail.2018.01.001

34. Floegel TA, Allen KD, Buman MP. A pilot study examining activity monitor use in older adults with heart failure during and after hospitalization. *Geriatr Nurs N Y N*. October 2018. doi:10.1016/j.gerinurse.2018.10.001

35. Shen H, Zhao J, Zhou X, et al. Impaired chronotropic response to physical activities in heart failure patients. *BMC Cardiovasc Disord*. 2017;17(1):136. doi:10.1186/s12872-017-0571-9

36. Mungovan SF, Singh P, Gass GC, Smart NA, Hirschhorn AD. Effect of physical activity in the first five days after cardiac surgery. *J Rehabil Med*. 2017;49(1):71-77. doi:10.2340/16501977-2165

37. Sweeting J, Ball K, McGaughran J, Atherton J, Semsarian C, Ingles J. Impact of the implantable cardioverter defibrillator on confidence to undertake physical activity in inherited heart disease: A cross-sectional study. *Eur J Cardiovasc Nurs J Work Group Cardiovasc Nurs Eur Soc Cardiol*. 2017;16(8):742-752. doi:10.1177/1474515117715760

38. Salmoirago-Blotcher E, Wayne PM, Dunsiger S, et al. Tai Chi Is a Promising Exercise Option for Patients With Coronary Heart Disease Declining Cardiac Rehabilitation. *J Am Heart Assoc*. 2017;6(10). doi:10.1161/JAHA.117.006603

39. McCarthy MM, Dickson VV, Katz SD, Chyun DA. An Exercise Counseling Intervention in Minority Adults With Heart Failure. *Rehabil Nurs Off J Assoc Rehabil Nurses*. 2017;42(3):146-156. doi:10.1002/rnj.265

40. Ter Hoeve N, Sunamura M, van Geffen ME, et al. Changes in Physical Activity and Sedentary Behavior During Cardiac Rehabilitation. *Arch Phys Med Rehabil*. 2017;98(12):2378-2384. doi:10.1016/j.apmr.2017.05.008

41. Christle JW, Schlumberger A, Haller B, Gloeckl R, Halle M, Pressler A. Individualized vs. group exercise in improving quality of life and physical activity in patients with cardiac disease and low exercise capacity: Results from the DOPPELHERZ trial. *Disabil Rehabil*. 2017;39(25):2566-2571. doi:10.1080/09638288.2016.1242174

42. Thorup CB, Andreasen JJ, Sørensen EE, Grønkjær M, Dinesen BI, Hansen J. Accuracy of a step counter during treadmill and daily life walking by healthy adults and patients with cardiac disease. *BMJ Open*. 2017;7(3):e011742. doi:10.1136/bmjopen-2016-011742

43. Waring T, Gross K, Soucier R, ZuWallack R. Measured Physical Activity and 30-Day Rehospitalization in Heart Failure Patients. *J Cardiopulm Rehabil Prev*. 2017;37(2):124-129. doi:10.1097/HCR.0000000000000204

44. Vogel J, Auinger A, Riedl R, Kindermann H, Helfert M, Ocenasek H. Digitally enhanced recovery: Investigating the use of digital self-tracking for monitoring leisure time physical activity of cardiovascular disease (CVD) patients undergoing cardiac rehabilitation. *PloS One*. 2017;12(10):e0186261. doi:10.1371/journal.pone.0186261

45. Nakajima M, Totsugawa T, Sakaguchi T, et al. Changes in the amount of physical activity in minimally invasive cardiac surgery. *J Phys Ther Sci*. 2017;29(11):2035-2038. doi:10.1589/jpts.29.2035

46. Alharbi M, Bauman A, Neubeck L, Gallagher R. Validation of Fitbit-Flex as a measure of free-living physical activity in a community-based phase III cardiac rehabilitation population. *Eur J Prev Cardiol*. 2016;23(14):1476-1485. doi:10.1177/2047487316634883

47. Ramadi A, Buijs DM, Threlfall TG, et al. Long-term Physical Activity Behavior After Completion of Traditional Versus Fast-track Cardiac Rehabilitation. *J Cardiovasc Nurs*. 2016;31(6):E1-E7. doi:10.1097/JCN.0000000000000341

48. Midence L, Arthur HM, Oh P, Stewart DE, Grace SL. Women’s Health Behaviours and Psychosocial Well-Being by Cardiac Rehabilitation Program Model: A Randomized Controlled Trial. *Can J Cardiol*. 2016;32(8):956-962. doi:10.1016/j.cjca.2015.10.007

49. Edwards MK, Loprinzi PD. Sedentary behavior & health-related quality of life among congestive heart failure patients. *Int J Cardiol*. 2016;220:520-523. doi:10.1016/j.ijcard.2016.06.256

50. Loprinzi PD, Addoh O. The Effects of Free-Living Physical Activity on Mortality After Coronary Artery Disease Diagnosis. *Clin Cardiol*. 2016;39(3):165-169. doi:10.1002/clc.22508

51. Melin M, Hagerman I, Gonon A, Gustafsson T, Rullman E. Variability in Physical Activity Assessed with Accelerometer Is an Independent Predictor of Mortality in CHF Patients. *PloS One*. 2016;11(4):e0153036. doi:10.1371/journal.pone.0153036

52. Charman SJ, van Hees VT, Quinn L, et al. The effect of percutaneous coronary intervention on habitual physical activity in older patients. *BMC Cardiovasc Disord*. 2016;16(1):248. doi:10.1186/s12872-016-0428-7

53. Prince SA, Blanchard CM, Grace SL, Reid RD. Objectively-measured sedentary time and its association with markers of cardiometabolic health and fitness among cardiac rehabilitation graduates. *Eur J Prev Cardiol*. 2016;23(8):818-825. doi:10.1177/2047487315617101

54. Granegger M, Schlöglhofer T, Ober H, Zimpfer D, Schima H, Moscato F. Daily life activity in patients with left ventricular assist devices. *Int J Artif Organs*. 2016;39(1):22-27. doi:10.5301/ijao.5000464

55. Thorup C, Hansen J, Grønkjær M, et al. Cardiac Patients’ Walking Activity Determined by a Step Counter in Cardiac Telerehabilitation: Data From the Intervention Arm of a Randomized Controlled Trial. *J Med Internet Res*. 2016;18(4):e69. doi:10.2196/jmir.5191

56. Kraal JJ, Sartor F, Papini G, et al. Energy expenditure estimation in beta-blocker-medicated cardiac patients by combining heart rate and body movement data. *Eur J Prev Cardiol*. 2016;23(16):1734-1742. doi:10.1177/2047487316667786

57. Izawa KP, Watanabe S, Hirano Y, et al. Gender-related differences in maximum gait speed and daily physical activity in elderly hospitalized cardiac inpatients: A preliminary study. *Medicine (Baltimore)*. 2015;94(11):e623. doi:10.1097/MD.0000000000000623

58. Alosco ML, Brickman AM, Spitznagel MB, et al. Daily Physical Activity Is Associated with Subcortical Brain Volume and Cognition in Heart Failure. *J Int Neuropsychol Soc JINS*. 2015;21(10):851-860. doi:10.1017/S1355617715000697

59. Alosco ML, Spitznagel MB, Cohen R, et al. Decreases in daily physical activity predict acute decline in attention and executive function in heart failure. *J Card Fail*. 2015;21(4):339-346. doi:10.1016/j.cardfail.2014.12.010

60. Redfield MM, Anstrom KJ, Levine JA, et al. Isosorbide Mononitrate in Heart Failure with Preserved Ejection Fraction. *N Engl J Med*. 2015;373(24):2314-2324. doi:10.1056/NEJMoa1510774

61. Frederix I, Van Driessche N, Hansen D, et al. Increasing the medium-term clinical benefits of hospital-based cardiac rehabilitation by physical activity telemonitoring in coronary artery disease patients. *Eur J Prev Cardiol*. 2015;22(2):150-158. doi:10.1177/2047487313514018

62. Frederix I, Hansen D, Coninx K, et al. Medium-Term Effectiveness of a Comprehensive Internet-Based and Patient-Specific Telerehabilitation Program With Text Messaging Support for Cardiac Patients: Randomized Controlled Trial. *J Med Internet Res*. 2015;17(7):e185. doi:10.2196/jmir.4799

63. Tang Y, Green P, Maurer M, et al. Relationship Between Accelerometer-Measured Activity and Self-Reported or Performance-Based Function in Older Adults with Severe Aortic Stenosis. *Curr Geriatr Rep*. 2015;4(4):377-384. doi:10.1007/s13670-015-0152-7

64. Nishitani-Yokoyama M, Miyauchi K, Shimada K, et al. Effects of Phase II Comprehensive Cardiac Rehabilitation on Coronary Plaque Volume After Acute Coronary Syndrome. *Int Heart J*. 2015;56(6):597-604. doi:10.1536/ihj.15-049

65. Dontje ML, van der Wal MHL, Stolk RP, et al. Daily physical activity in stable heart failure patients. *J Cardiovasc Nurs*. 2014;29(3):218-226. doi:10.1097/JCN.0b013e318283ba14

66. Devi R, Powell J, Singh S. A web-based program improves physical activity outcomes in a primary care angina population: Randomized controlled trial. *J Med Internet Res*. 2014;16(9):e186. doi:10.2196/jmir.3340

67. Evenson KR, Butler EN, Rosamond WD. Prevalence of physical activity and sedentary behavior among adults with cardiovascular disease in the United States. *J Cardiopulm Rehabil Prev*. 2014;34(6):406-419. doi:10.1097/HCR.0000000000000064

68. Fulcher KK, Alosco ML, Miller L, et al. Greater physical activity is associated with better cognitive function in heart failure. *Health Psychol Off J Div Health Psychol Am Psychol Assoc*. 2014;33(11):1337-1343. doi:10.1037/hea0000039

69. Oliveira NL, Ribeiro F, Teixeira M, et al. Effect of 8-week exercise-based cardiac rehabilitation on cardiac autonomic function: A randomized controlled trial in myocardial infarction patients. *Am Heart J*. 2014;167(5):753-761.e3. doi:10.1016/j.ahj.2014.02.001

70. Alosco ML, Spitznagel MB, Cohen R, et al. Decreased physical activity predicts cognitive dysfunction and reduced cerebral blood flow in heart failure. *J Neurol Sci*. 2014;339(1-2):169-175. doi:10.1016/j.jns.2014.02.008

71. Byun W, Ozemek C, Riggin K, Strath S, Kaminsky L. Correlates of objectively measured physical activity in cardiac patients. *Cardiovasc Diagn Ther*. 2014;4(5):406-410. doi:10.3978/j.issn.2223-3652.2014.10.06

72. Borland M, Rosenkvist A, Cider A. A group-based exercise program did not improve physical activity in patients with chronic heart failure and comorbidity: A randomized controlled trial. *J Rehabil Med*. 2014;46(5):461-467. doi:10.2340/16501977-1794

73. Izawa KP, Watanabe S, Oka K, et al. Association between mental health and physical activity in patients with chronic heart failure. *Disabil Rehabil*. 2014;36(3):250-254. doi:10.3109/09638288.2013.785604

74. Klompstra L, Jaarsma T, Strömberg A. Exergaming to increase the exercise capacity and daily physical activity in heart failure patients: A pilot study. *BMC Geriatr*. 2014;14:119. doi:10.1186/1471-2318-14-119

75. da Silva VZM, Lima AC, Vargas FT, Cahalin LP, Arena R, Cipriano G. Association between physical activity measurements and key parameters of cardiopulmonary exercise testing in patients with heart failure. *J Card Fail*. 2013;19(9):635-640. doi:10.1016/j.cardfail.2013.08.002

76. Hu SXH, Keogh AM, Macdonald PS, et al. Interaction between physical activity and continuous-flow left ventricular assist device function in outpatients. *J Card Fail*. 2013;19(3):169-175. doi:10.1016/j.cardfail.2013.01.008

77. Bäck M, Cider A, Gillström J, Herlitz J. Physical activity in relation to cardiac risk markers in secondary prevention of coronary artery disease. *Int J Cardiol*. 2013;168(1):478-483. doi:10.1016/j.ijcard.2012.09.117

78. Kaminsky LA, Jones J, Riggin K, Strath SJ. A pedometer-based physical activity intervention for patients entering a maintenance cardiac rehabilitation program: A pilot study. *Cardiovasc Diagn Ther*. 2013;3(2):73-79. doi:10.3978/j.issn.2223-3652.2013.03.03

79. Mohri M, Motohama R, Sato N. Home-based cardiac rehabilitation decreases red cell distribution width in chronic heart failure. *Acta Cardiol*. 2013;68(6):615-619. doi:10.2143/AC.68.6.8000009

80. Izawa KP, Watanabe S, Oka K, et al. Usefulness of step counts to predict mortality in Japanese patients with heart failure. *Am J Cardiol*. 2013;111(12):1767-1771. doi:10.1016/j.amjcard.2013.02.034

81. Sherwood A, Bower JK, Routledge FS, et al. Nighttime blood pressure dipping in postmenopausal women with coronary heart disease. *Am J Hypertens*. 2012;25(10):1077-1082. doi:10.1038/ajh.2012.95

82. Karjalainen JJ, Kiviniemi AM, Hautala AJ, et al. Effects of exercise prescription on daily physical activity and maximal exercise capacity in coronary artery disease patients with and without type 2 diabetes. *Clin Physiol Funct Imaging*. 2012;32(6):445-454. doi:10.1111/j.1475-097X.2012.01148.x

83. Alosco ML, Spitznagel MB, Miller L, et al. Depression is associated with reduced physical activity in persons with heart failure. *Health Psychol Off J Div Health Psychol Am Psychol Assoc*. 2012;31(6):754-762. doi:10.1037/a0028711

84. Izawa KP, Watanabe S, Oka K, et al. Relation between physical activity and exercise capacity of ≥5 metabolic equivalents in middle- and older-aged patients with chronic heart failure. *Disabil Rehabil*. 2012;34(23):2018-2024. doi:10.3109/09638288.2012.667502

85. Houle J, Doyon O, Vadeboncoeur N, Turbide G, Diaz A, Poirier P. Effectiveness of a pedometer-based program using a socio-cognitive intervention on physical activity and quality of life in a setting of cardiac rehabilitation. *Can J Cardiol*. 2012;28(1):27-32. doi:10.1016/j.cjca.2011.09.020

86. Guiraud T, Granger R, Gremeaux V, et al. Telephone support oriented by accelerometric measurements enhances adherence to physical activity recommendations in noncompliant patients after a cardiac rehabilitation program. *Arch Phys Med Rehabil*. 2012;93(12):2141-2147. doi:10.1016/j.apmr.2012.06.027

87. Izawa KP, Watanabe S, Hiraki K, et al. Determination of the effectiveness of accelerometer use in the promotion of physical activity in cardiac patients: A randomized controlled trial. *Arch Phys Med Rehabil*. 2012;93(11):1896-1902. doi:10.1016/j.apmr.2012.06.015

88. Reid RD, Morrin LI, Beaton LJ, et al. Randomized trial of an internet-based computer-tailored expert system for physical activity in patients with heart disease. *Eur J Prev Cardiol*. 2012;19(6):1357-1364. doi:10.1177/1741826711422988

89. Reid RD, Morrin LI, Higginson LAJ, et al. Motivational counselling for physical activity in patients with coronary artery disease not participating in cardiac rehabilitation. *Eur J Prev Cardiol*. 2012;19(2):161-166. doi:10.1177/1741826711400519

90. Pinto BM, Goldstein MG, Papandonatos GD, et al. Maintenance of exercise after phase II cardiac rehabilitation: A randomized controlled trial. *Am J Prev Med*. 2011;41(3):274-283. doi:10.1016/j.amepre.2011.04.015

91. Ribeiro F, Alves AJ, Teixeira M, et al. Exercise training increases interleukin-10 after an acute myocardial infarction: A randomised clinical trial. *Int J Sports Med*. 2012;33(3):192-198. doi:10.1055/s-0031-1297959

92. Houle J, Doyon O, Vadeboncoeur N, Turbide G, Diaz A, Poirier P. Innovative program to increase physical activity following an acute coronary syndrome: Randomized controlled trial. *Patient Educ Couns*. 2011;85(3):e237-244. doi:10.1016/j.pec.2011.03.018

93. Tai M-K, Meininger JC, Frazier LQ, Chan W. Ambulatory blood pressure and physical activity in heart failure. *Biol Res Nurs*. 2010;11(3):269-279. doi:10.1177/1099800409337731

94. Toth MJ, Shaw AO, Miller MS, et al. REDUCED KNEE EXTENSOR FUNCTION IN HEART FAILURE IS NOT EXPLAINED BY INACTIVITY. *Int J Cardiol*. 2010;143(3):276-282. doi:10.1016/j.ijcard.2009.02.040

95. Barnason S, Zimmerman L, Schulz P, Tu C. Influence of an early recovery telehealth intervention on physical activity and functioning after coronary artery bypass surgery among older adults with high disease burden. *Heart Lung J Crit Care*. 2009;38(6):459-468. doi:10.1016/j.hrtlng.2009.01.010

96. Stevenson TG, Riggin K, Nagelkirk PR, Hargens TA, Strath SJ, Kaminsky LA. Physical activity habits of cardiac patients participating in an early outpatient rehabilitation program. *J Cardiopulm Rehabil Prev*. 2009;29(5):299-303. doi:10.1097/HCR.0b013e3181b4ca61

97. Brändström Y, Brink E, Grankvist G, Alsén P, Herlitz J, Karlson BW. Physical activity six months after a myocardial infarction. *Int J Nurs Pract*. 2009;15(3):191-197. doi:10.1111/j.1440-172X.2009.01744.x

98. Butler L, Furber S, Phongsavan P, Mark A, Bauman A. Effects of a pedometer-based intervention on physical activity levels after cardiac rehabilitation: A randomized controlled trial. *J Cardiopulm Rehabil Prev*. 2009;29(2):105-114. doi:10.1097/HCR.0b013e31819a01ff

99. Maurer MS, Cuddihy P, Weisenberg J, et al. The prevalence and impact of anergia (lack of energy) in subjects with heart failure and its associations with actigraphy. *J Card Fail*. 2009;15(2):145-151. doi:10.1016/j.cardfail.2008.10.021

100. Oliveira J, Ribeiro F, Gomes H. Effects of a home-based cardiac rehabilitation program on the physical activity levels of patients with coronary artery disease. *J Cardiopulm Rehabil Prev*. 2008;28(6):392-396. doi:10.1097/HCR.0b013e31818c3b83

101. Papaspyros S, Uppal S, Khan SA, Paul S, O’Regan DJ. Analysis of bedside entertainment services’ effect on post cardiac surgery physical activity: A prospective, randomised clinical trial. *Eur J Cardio-Thorac Surg Off J Eur Assoc Cardio-Thorac Surg*. 2008;34(5):1022-1026. doi:10.1016/j.ejcts.2008.05.042

102. Ayabe M, Brubaker PH, Dobrosielski D, et al. Target step count for the secondary prevention of cardiovascular disease. *Circ J Off J Jpn Circ Soc*. 2008;72(2):299-303. doi:10.1253/circj.72.299

103. Hughes AR, Mutrie N, Macintyre PD. Effect of an exercise consultation on maintenance of physical activity after completion of phase III exercise-based cardiac rehabilitation. *Eur J Cardiovasc Prev Rehabil Off J Eur Soc Cardiol Work Groups Epidemiol Prev Card Rehabil Exerc Physiol*. 2007;14(1):114-121. doi:10.1097/HJR.0b013e3280116485

104. Jones NL, Schneider PL, Kaminsky LA, Riggin K, Taylor AM. An assessment of the total amount of physical activity of patients participating in a phase III cardiac rehabilitation program. *J Cardiopulm Rehabil Prev*. 2007;27(2):81-85. doi:10.1097/01.HCR.0000265034.39404.07

105. Evangelista LS, Dracup K, Doering L, Moser DK, Kobashigawa J. Physical activity patterns in heart transplant women. *J Cardiovasc Nurs*. 2005;20(5):334-339.

106. van den Berg-Emons R, Balk A, Bussmann H, Stam H. Does aerobic training lead to a more active lifestyle and improved quality of life in patients with chronic heart failure? *Eur J Heart Fail*. 2004;6(1):95-100. doi:10.1016/j.ejheart.2003.10.005

107. Izawa KP, Yamada S, Oka K, et al. Long-term exercise maintenance, physical activity, and health-related quality of life after cardiac rehabilitation. *Am J Phys Med Rehabil*. 2004;83(12):884-892.

108. Ayabe M, Brubaker PH, Dobrosielski D, et al. The physical activity patterns of cardiac rehabilitation program participants. *J Cardpulm Rehabil*. 2004;24(2):80-86.
